# Retrograde Procedural Memory in Parkinson’s Disease: A Cross-Sectional, Case-Control Study

**DOI:** 10.3233/JPD-213081

**Published:** 2022-04-05

**Authors:** Laure Pauly, Claire Pauly, Maxime Hansen, Valerie E. Schröder, Armin Rauschenberger, Anja K. Leist, Rejko Krüger, on behalf of the NCER-PD Consortium

**Affiliations:** a Transversal Translational Medicine, Luxembourg Institute of Health, Strassen, Luxembourg; b Faculty of Science, Technology and Medicine, University of Luxembourg, Esch-sur-Alzette, Luxembourg; c Luxembourg Centre for Systems Biomedicine, University of Luxembourg, Esch-sur-Alzette, Luxembourg; d Department of Neurology, Centre Hospitalier de Luxembourg, Strassen, Luxembourg; e Department of Social Sciences, Institute for Research on Socio-Economic Inequality, University of Luxembourg, Esch-sur-Alzette, Luxembourg

**Keywords:** Parkinson’s disease, neurodegenerative disorder, cognitive impairment, memory, habits, neuropsychology

## Abstract

**Background:**

The analysis of the procedural memory is particularly relevant in neurodegenerative disorders like Parkinson’s disease, due to the central role of the basal ganglia in procedural memory. It has been shown that anterograde procedural memory, the ability to learn a new skill, is impaired in Parkinson’s disease. However, retrograde procedural memory, the long-term retention and execution of skills learned in earlier life stages, has not yet been systematically investigated in Parkinson’s disease.

**Objective:**

This study aims to investigate retrograde procedural memory in people with Parkinson’s disease. We hypothesized that retrograde procedural memory is impaired in people with Parkinson’s disease compared to an age- and gender-matched control group.

**Methods:**

First, we developed the CUPRO evaluation system, an extended evaluation system based on the Cube Copying Test, to distinguish the cube copying procedure, representing functioning of retrograde procedural memory, and the final result, representing the visuo-constructive abilities. Development of the evaluation system included tests of discriminant validity.

**Results:**

Comparing people with typical Parkinson’s disease (*n* = 201) with age- and gender-matched control subjects (*n* = 201), we identified cube copying performance to be significantly impaired in people with Parkinson’s disease (*p* = 0.008). No significant correlation was observed between retrograde procedural memory and disease duration.

**Conclusion:**

We demonstrated lower cube copying performance in people with Parkinson’s disease compared to control subjects, which suggests an impaired functioning of retrograde procedural memory in Parkinson’s disease.

## INTRODUCTION

Many daily life activities such as driving a car, tying one’s shoes, or typing on the computer rely on procedural learning and its automation, the procedural memory. Given that its impaired functioning is linked with significant distress, we must deepen our understanding of this memory concept. This implicit, long-term memory stores information on unconscious cognitive or motor procedures. Procedural memory is characterized by its robustness and its capacity to maintain knowledge over a long period of time, even if it is not regularly consolidated. It is typically acquired through repetition, characterized by an improvement in performance, followed by automatization of the skill [1]. Automatization is reached when the neural network involved in performing the task can execute it without the need for conscious thought [2].

Brenda Milner [3], one of the pioneers in the field of cognitive neurosciences, provided the first solid evidence of spatial and conceptual dissociation of explicit versus implicit memory. She made major contributions to the understanding of the memory systems, among others the procedural memory. Whereas declarative memory appears to be dependent on the medial temporal lobe and the diencephalic structures, the most important brain components involved in the formation and consolidation of non-declarative, procedural memory are the basal ganglia, especially the striatum [4–7].

Procedural memory can be separated into an ante-rograde and a retrograde component. The anterograde procedural memory involves the acquisition of new skills, whereas the ability to execute skills acquired in earlier life stages is part of retrograde procedural memory [8]. Observations on retrograde procedural memory have been done indirectly in form of case-reports [9] and studies on musical memory or overlearned language (e.g., songs, poems) [8, 10]. However, to our knowledge, validated protocols are missing to evaluate the very long-term retention and retrieval of contents in procedural memory, the retrograde part of the memory concept.

Therefore, we developed a brief and easy to administer assessment tool that allows to evaluate the functioning of retrograde procedural memory. Based on the Cube Copying Test, also called Necker’s Cube [11], we established an extended evaluation system that assesses both the copying procedure, representing retrograde procedural memory, and the final result, representing visuo-constructive functions. The Cube Copying Test, is a short screening tool, widely used in clinical and research settings. It is incorporated in commonly used assessments like the Montreal Cognitive Assessment (MoCA) screening test [12] and the Consortium to Establish a Registry for Alzheimer’s Disease (CERAD) neuropsychological battery [13]. The Cube Copying Test is typically applied to evaluate visuo-constructive cognitive function or constructional praxis, associated with visuo-spatial disorders which are characterized by an impairment in the spatial organization necessary to assemble individual parts to a single entity.

We applied this extended evaluation system of the Cube Copying Test, that we named CUPRO evaluation system (short for CUbe drawing PROcedure), on people with typical Parkinson’s disease, as this disease is characterized by a loss of dopaminergic innervation in the basal ganglia and as the basal ganglia play a central role in procedural memory [4]. Despite the importance of procedural memory in our daily life activities and the numerous studies that have investigated this topic, there are still many discrepancies. These controversies are mainly due to the varying definitions of the memory concept and to the nature of the used tasks [14]. Until now, assessments primarily evaluated the motor, perceptual and cognitive procedural learning, with tasks such as the pursuit rotor task [15, 16], serial reaction time task [17, 18], and arithmetic alphabet test [19].

Only few studies focused on the suggested long-term retention of new skills (3–18 months) [7, 20, 21]. As mentioned by Cohen [20] “surprisingly little work has specifically looked at how and whether this learning is maintained in the long-term. Results, which indicate that a new skill information is retained over a testing period, provide no evidence that learning will be retained over a longer period of time”.

To our knowledge, this study is the first assessing the very long-term retention and retrieval of contents in procedural memory, that have been learned in earlier life stages in a cohort of deeply phenotyped people with Parkinson’s disease [22]. Investigating retrograde procedural memory in Parkinson’s disease increases our understanding of the disease’s cognitive profile. Gaining insights on impairments in retrograde procedural memory may in the long run even contribute to the treatment of symptoms of Parkinson’s disease, since the inability to carry out procedural tasks may have its roots in impaired procedural memory functioning.

The main objectives of our study were, firstly, to develop a tool to assess functioning of retrograde procedural memory by extending the evaluation system of the Cube Copying Test. The development of this CUPRO evaluation system included tests of discriminant validity, given that a wide range of cognitive and neural processing capabilities are required for accurate cube copying [23]. The second objective was to validate the hypothesis of a deficit of retrograde procedural memory in people with Parkinson’s disease compared with control subjects. We hypothesized that people with Parkinson’s disease may have more difficulties recalling an acquired copying procedure of the cube than the control subjects, thereby evaluating two components of the Cube Copying Test, the procedure of copying the cube and the correctness of the outcome. To gain further insights into the functioning of retrograde procedural memory, we additionally explored associations between cube copying performance and disease characteristics.

## MATERIAL AND METHODS

### Participants

All participants were recruited from the Luxem-bourg Parkinson’s Study of the National Centre of Excellence in Research on Parkinson’s disease (NCER-PD), a monocentric, observational, longitudinal prospective study with annual follow-ups of people with Parkinson’s disease and a control group from Luxembourg and the Greater Region [22]. All participants provided informed consent according to the Declaration of Helsinki. The study was approved by the National Ethics Board (CNER Ref: 201407/13).

In the present study, 402 participants were enrolled, including 201 people with Parkinson’s disease and 201 control subjects. Diagnosis of typical Parkinson’s disease was based on the United Kingdom Parkinson’s Disease Society Brain Bank Clinical Diagnostic Criteria [24]. Each subject underwent a detailed neurological examination and provided information on early symptoms, disease history and treatment. Patients were tested while being on their regular medication. Levodopa Equivalent Daily Dose (LEDD) was calculated for each participant according to Tomlinson [25]. The Unified Parkinson’s disease Rating Scale MDS-UPDRS-III [26] and the Hoehn and Yahr scale [27] were used to assess motor symptoms and disease stage. Inclusion criteria were age 18 years or older and ability to sign the written informed consent. Excluded were people with Parkinson’s disease having undergone brain surgery (i.e., deep brain stimulation) or having been diagnosed with Parkinson’s disease with dementia (as defined in [28]), atypical forms of parkinsonism, as well as other neurological diseases. Participants with a history of severe psychiatric disorders (e.g., schizophrenia) or traumatic brain injury were also excluded.

### Developing an extended evaluation system of the Cube Copying Test, the CUPRO evaluation system

The Cube Copying Test was initially evaluated with the classical scoring system established by Nasreddine and colleagues [12]. One point was given for a correct final result: Drawing must be three-dimensional; the orientation of the drawing must be correct; the final result must be correct (i.e., no line is added/missing, lines are relatively parallel, length of lines is relatively similar). The point was not given if any of these criteria was not met.

Until now, only unsystematic observations in form of case reports [9] or studies on musical memory or overlearned language [8, 10] point to a potential deficit of retrograde procedural memory in Parkinson’s disease. Before establishing this study topic, we repeatedly observed that a lot of people with Parkinson’s disease applied unexpected procedures for copying the cube in the MoCA test [12], which is part of the neuropsychological test battery. Drawing geometric forms is taught in primary school [29], so it is reasonable to assume that this skill has been acquired in participants with completed primary education. The Cube Copying Test meets the conditions of assessing retrograde procedural memory: by copying the cube, a (i) previously learned procedure is (ii) unconsciously applied.

**Fig. 1:**
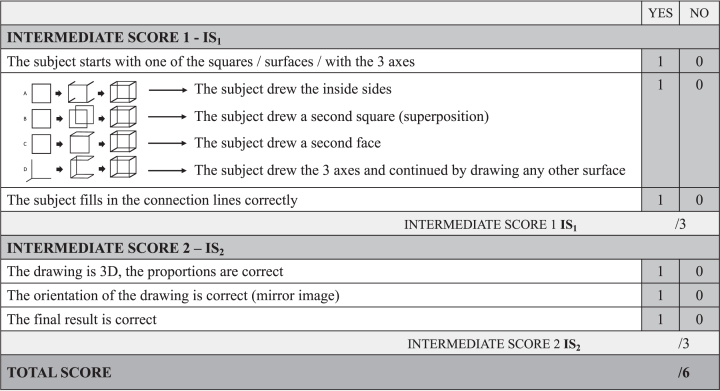
Representation of the CUPRO evaluation system, an extended evaluation system for the Cube Copying Test. The first intermediate score (IS_1_) evaluates the copying procedure, the second intermediate score (IS_2_) the visuo-constructive functions. A-D) Representation of the four copying procedures.

During a pilot study on a group of control subjects (*n* = 40), four recurrent procedures were identified as representative patterns and are referred to as “typical” procedures in the following (Fig. 1A–D).

For the procedures A, B, and C, the copying begins with the drawing of one of the six faces of the cube. Then the copying is differentiated into three possible procedures: The subject continues by A. drawing the sidelines backwards/forwards; by B. superimposing a second square; by C. drawing any second face of the cube. For the procedure D, the copying begins with drawing lines similar to the coordinate axes in three-dimensional space (x,y,z). The drawing is completed as soon as all the elements are connected. Similar observations on standard cube drawing strategies were also made by van Sommers [30, 31].

As a first step, we extended this scoring system to separately assess whether the drawing is three-dim-ensional (1 point), if the orientation of the drawing is correct (1 point), and if the final result is correct (1 point) (Fig. 1 –Intermediate Score 2 (IS_2_)). Sub-sequently, the Cube Copying Test was further extended to additionally evaluate the copying procedure itself. Based on the four typical procedures observed, the extended scoring system evaluates the starting approach; 1 point is administered if the subject started with one of the squares/surfaces/with the 3 axes. Further, the procedure itself is evaluated on 1 point (A.-D.). The last point is administered if the subject accomplished the copying procedure, by connecting the lines (Fig. 1 –Intermediate Score 1 (IS_1_)).

The total score of six points of the CUPRO evaluation system is composed of two intermediate scores. The first intermediate score on three points (IS_1_) evaluates the copying procedure. The second intermediate score (IS_2_) of three points allows us to infer aspects related to visuo-constructive functions.

For the copying of the cube, a sheet of paper was placed in front of the participant. The participant was asked to copy the drawing as accurately as possible. The drawing procedure was evaluated unbeknownst to the subject to ensure that the copying performance did not depend on explicit memory processes. No time limit was imposed. The tests were administered by a trained neuropsychologist or research nurse and scored according to the procedure described above.

### Neuropsychological assessments

The global cognitive function was evaluated with the Montreal Cognitive Assessment [12], part of the basic assessment level (Level A). An optional assessment level (Level B) including a variety of other neuropsychological assessments was also proposed to the participants [22]. This level included inter alia, the Judgment of Line Orientation test used for measuring of visuospatial judgment [32], the Qualitative Scoring MMSE Pentagon test for the visuo-constructive abilities [33], the Complex Rey Figure for the visuo-constructive and planning functions [34] and the Frontal Assessment Battery for the assessment of executive functions [35].

### Self-assessment questionnaires

Three different self-rating questionnaires were used: the Beck Depression Inventory (BDI-I) questionnaire [36], the Starkstein Apathy Scale (SAS) [37] and the Parkinson’s Disease Questionnaire (PDQ-39) [38] to assess the presence of depression symptomatology, apathy, and quality of life in people with Parkinson’s disease, respectively.

### Statistics

The two groups were matched by age and gender by Propensity Score Matching (matching tolerance = 0.05). Differences in demographic and clinical characteristics as well as the cube performance differences between the groups were analyzed using the non-parametric Mann-Whitney U test and Pearson’s chi-squared test (two-tailed). Correlations were tested with the bivariate Spearman correlation test. The significance threshold was set at *p*≤0.05. The *p*-values were assessed for significance using a Bonferroni corrected significance level. All statistical analyses were performed using RStudio version 1.3.1093 (RRID:SCR_000432; R Version 4.0.3 (2020-10-10)).

### Data availability statement

All supporting material, data and software are available here: https://doi.org/10.17881/7bwb-aj16.

## RESULTS

For statistically significant results, we report the estimated correlation coefficients (Spearman correlation test), the observed percentages (Pearson’s chi-squared test), and the mean difference between groups (Mann-Whitney U test).

Confirming successful matching, the groups did not differ significantly in gender (*p* = 0.920), age (*p* = 0.943), years of education (*p* = 0.128), handedness (*p* = 0.139), and MoCA score (*p* = 0.246). As expected, people with Parkinson’s disease presented significantly higher scores on the BDI-I (MD = 3.37, *p* < 0.001), the SAS (MD = 3.79, *p* < 0.001), and the MDS-UPDRS-III (MD = 28.21, *p* < 0.001) compared to the control subjects. Concerning number of languages spoken, people with Parkinson’s disease spoke significantly fewer languages than the control subjects (MD = –0.75, *p* < 0.001) (Table 1).

**Table 1:** Demographic and clinical data for people with Parkinson’s disease (*n* = 201) and control subjects (*n* = 201)

	Descriptive statistics						*p*
Variable	PD (*n* = 201)			CS (*n* = 201)			PD vs. CS
	*Mean*	*SD*	*Range*	*Mean*	*SD*	*Range*
Gender, M / F	111 / 90		–	109 / 92		–	0.920
Handedness, R / L / A	170 / 14/ 7^+10na^		–	180/6/10^+5na^		–	0.139
Age, y	64.84	10.20	22–87	64.71	10.18	30–86	0.943
Education, y	13.60	3.80	4–25	14.25	3.96	4–24	0.128
MOCA total score (/30)	26.58	2.68	21–30	26.97	2.29	21–30	0.246
MDS-UPDRS-III (/132)	32.80	13.40	7–88	4.59	5.10	0–27	< 0.001^*,**^
Hoehn and Yahr	2.06	0.53	0.00	0.00	< 0.001^*,**^
Stage 1 / 1.5 / 2 /	19 / 13 / 119 / 29 /	
2.5 / 3 / 4 / 5	18 / 2 / 0+^1na^	
BDI-I (/63)	8.32	6.36	0–34	4.95	4.72	0–27	< 0.001^*,**^
SAS (/42)	13.63	5.49	1–32	9.84	4.75	0–25	< 0.001^*,**^
Languages spoken	2.81	1.10	1–4	3.56	0.78	1–4	< 0.001^*,**^
Disease duration, y	5.37	4.39	0–24	–	–	–	–
LEDD	596.35	391.30	50–2062	–	–	–	–

SD, standard deviation; PD, people with Parkinson’s disease; CS, control subjects; M, male; F, female; R, right-handed; L, left-handed; A, ambidextrous; na, not available; n, sample size; MDS-UPDRS, Movement Disorder Society - Unified Parkinson’s Disease Rating Scale; BDI, Beck Depression Inventory; SAS, Starkstein Apathy Scale; MoCA, Montreal Cognitive Assessment; LEDD, Levodopa Equivalent Daily Dose. ^*^Significant at the 5% level (2-tailed). ^**^Significant at the Bonferroni-adjusted 5% level (*p* < = 0.05/10).

Within the PD group, those with impaired retrograde procedural memory were significantly older (MD = 4.18, *p* = 0.009), lower educated (MD = –1.08, *p* = 0.023), more likely to be female (54.43% versus 38.52%, *p* = 0.039), and had lower MoCA scores (MD = –1.39, *p* < 0.001) compared with those with unimpaired retrograde procedural memory. No significant differences on motor symptoms, LEDD, and disease duration were observed.

Group differences were found in the total score of the cube copy in both classical and extended evaluation system of the Cube Copying test: According to the classical evaluation system, people with Parkinson’s disease had a significantly lower average score than the control subjects (*p* < 0.001). With the extended evaluation system (CUPRO), people with Parkinson’s disease had significantly lower IS_1_ (MD = –0.38, *p* = 0.008) and IS_2_ scores (MD = –0.33, *p* = 0.013) than the control subjects. Investigating the differences in IS_1_ scores, we took a closer look at the distribution of the use and non-use of the pre-defined procedures (Table 2).

**Table 2:** Cube Scoring according to the classical evaluation (evaluated with one point) and extended evaluation system of the cube (evaluated with six points; divided into two intermediate scores: IS_1_ (assesses retrograde procedural memory) and IS_2_ (assesses the visuo-constructive functions)

		Descriptive statistics				*p*
	Variable	PD (*n* = 201)		CS (*n* = 201)		PD vs. CS
		*Mean*	*SD*	*Mean*	*SD*
Extended evaluation	IS_1_ (/3)	2.05	1.13	2.43	0.90	0.008^*,**^
system of the Cube	IS_2_ (/3)	2.26	1.10	2.59	0.84	0.013^*,**^
Copying Test
Classical evaluation score	% of participants	*Mean*	*Mean*
of the Cube Copying	with correct	65.67	83.58	< 0.001^*,**^
test (Nasreddine et al.)	result
[12]

The Cube Copying total score (classical evaluation system) on one point evaluates the final result of the cube; one point is administered if the copy is identical to the model. In the extended evaluation system: the first intermediate score (IS_1_) evaluates the drawing procedure. The second intermediate score (IS_2_) evaluates visuo-constructive functions. SD, standard deviations; PD, participants with Parkinson’s disease; CS, control subjects; IS, intermediate score. ^*^Significant at the 5% level (2-tailed). ^**^Significant at the Bonferroni-adjusted 5% level (*p* < = 0.05/3).

In people with Parkinson’s disease, age and quality of life were negatively correlated with retrograde procedural memory performance (IS_1_) (R = –0.228; *p* = 0.001 and R = –0.173; *p* = 0.018). Furthermore, higher MoCA scores and education were associated with a better retrograde procedural memory (R = +0.364, *p* < 0.001 and R = +0.224; *p* = 0.002). We found no significant correlation between IS_1_ and disease duration (R = –0.093; *p* = 0.216), IS_1_ and MDS-UPDRS-III score (R = –0.108; *p* = 0.129), IS_1_ and LEDD (R = +0.015; *p* = 0.842) and IS_1_ and depressive symptoms (R = –0.128; *p* = 0.075) (Table 3).

**Table 3:** Correlations for the Intermediate Scores 1 in the PD and the CS group

	Spearman Correlations					
	PD (*n* = 201)			CS (*n* = 201)		
	Spearman –Correlation coefficient R		*p*	Spearman –Correlation coefficient R		*p*
Disease Duration	–0.093		0.216	–	–	–
MDS-UPDRS-III	–0.108		0.129	–0.225	^*^	0.010
LEDD	+0.015		0.842	–	–	–
Education	+0.224	^*,^^**^	0.002	+0.106		0.135
MoCA total score	+0.364	^*a, **^	< 0.001	+0.203	^*^	0.004
Age	–0.228	^*a^	0.001	–0.006		0.931
BDI-I	–0.128		0.075	–0.060		0.404
SAS	–0.189	^*^	0.009	–0.092		0.201
Hoehn and Yahr	–0.150	^*^	0.035	–	–	–
PDQ-39	–0.173	^*^	0.018	–	–	–

PD, people with Parkinson’s disease; CS, vontrol subjects; UPDRS, Unified Parkinson’s Disease Rating Scale; LEDD, Levodopa Equivalent Daily Dose; MoCA, Montreal Cognitive Assessment; BDI, Beck Depression Inventory; SAS, Starkstein Apathy Scale; PDQ-39, Parkinson’s Disease Questionnaire –39 items. ^*^Significant at the 5% level (2-tailed). ^*a^Significant at the 1% level (2-tailed). ^**^Significant at the Bonferroni-adjusted 5% level (*p* < = 0.05/16).

Additional testing for discriminant validity by investigating associations of cube copying performance with several related constructs was done with a subgroup of participants for which relevant tests were available (34≤*N*≤73). Neither visuo-constru-ctive, visuo-spatial, planning nor executive functions significantly interfered with the score representing retrograde procedural memory (Supplementary Material).

## DISCUSSION

### Summary of findings

By developing and applying a new rating system of the Cube Copying Test, we demonstrated that people with Parkinson’s disease showed a lower cube copying performance compared to control subjects, which suggests an impaired functioning of retrograde procedural memory in Parkinson’s disease. The intermediate score, representing the procedure of cube copying (IS_1_), as a surrogate for functioning of cognitive retrograde procedural memory, was significantly reduced in people with Parkinson’s disease compared to age- and gender-matched controls (Table 2). The intermediate score could thus discriminate between people with and without Parkinson’s disease, reflecting known-group validity. Furthermore, our results support previous studies which assessed retention three to 18 months after learning of a new skill: people with Parkinson’s disease were less efficient than control subjects in maintaining skills over time [7, 20, 21]. In comparison with the control group, the patient group presented impaired visuo-constructive functions, in line with previous findings on Parkinson’s disease [39].

Elevated levels of depression, assessed by BDI-I, were observed between patients and control subjects at baseline. This observation at baseline is not unexpected, as depression is found in approximately 30–40% of people with Parkinson’s disease and may even precede motor symptoms [40]. Interestingly however, deficits in retrograde procedural memory in people with Parkinson’s disease were not correlated with symptoms of depression. Contrary to what might have been expected, no significant correlation was observed between retrograde procedural memory performance and the disease severity, defined by LEDD, MDS-UPDRS-III score, and disease duration, in Parkinson’s disease patients.

The significant correlation, observed between retrograde procedural memory and quality of life in people with Parkinson's disease, highlights the importance of investigating this memory.

Within the Parkinson’s disease patients, people with impaired retrograde procedural memory were more likely to be female, older, lower educated, and had lower cognitive performance than those with unimpaired retrograde procedural memory. Women may be more likely to show impairments on retrograde procedural memory due to lower visuo-spatial skills [41]. In research on Parkinson’s disease, education has been shown to predict lower risk of cognitive decline in Parkinson’s disease [42].

### Strengths and limitations

The new extended evaluation system was tested in a comparatively large sample of people with Parkinson’s disease and age- and gender-matched controls, and excluded several alternative explanations of impaired functioning of retrograde procedural memory by testing and controlling for a set of confounders.

Our evaluation system has a number of strengths, such as specifically assessing recall of previously learned procedures. As it is simple and easy to administer, it can be evaluated by any trained health professional. The time required for the CUPRO evaluation system is short (< 1 minute), and once familiar with it, the examiner can grade the cube copying performance, while simultaneously observing the subject during copying the figure. The Cube Copying Test is widely used in clinical and research settings and is already incorporated in standard assessments, i.e., in the MoCA Screening test. Therefore, the CUPRO evaluation system can be easily integrated without the need to include a new test. It adds valuable information to an already well-established screening tool without increasing the burden for patients. Furthermore, the novel test has potential for wide application, filling the gap of techniques to reliably assess functioning of retrograde procedural memory in clinical settings and giving valuable perspectives for future research. Moreover, for the evaluation of retrograde procedural memory, we focused on the procedure and not on the final result of the cube drawing. As such, it does not directly involve motor components, contrary to most of the already existing procedural memory tasks [43].

Through evaluating discriminant validity with several tests representing related constructs, we could not find evidence that motor deficits such as tremor and rigidity prevalent in Parkinson’s disease as well as deficits in visuo-constructive, visuo-spatial, planning or executive functions interfered with cube copying performance, further consolidating the value of the new extended evaluation. However, these results thus need to be interpreted with caution, as the absence of significant correlation could also be explained by low statistical power due to the small sub-sample.

A possible bias could be related to socio-cultural components, given that Luxembourg is characterized by a multinational society. However, after verification, no significant difference was observed in the intermediate score 1 for participants from geographical Europe in comparison to participants from other regions.

Indeed, how a cube is drawn is part of the primary or lower secondary school curriculum [44]. Schooling curricula may have differed across countries; however, anecdotal evidence from neighboring countries, suggests similarities of the timing when cube drawing is taught at school. Regarding the current Luxembourgish school program, the drawing of geometric figures is scheduled at latest in the 6th year of schooling [29]. According to the study conducted by Cox [45] six years of education are sufficient for participants to know how to draw a cube. In this study, most of the participants (98.5%) had a duration of education of ≥6 years, consistent with rates of lower secondary education completion in many developed countries over the last decades. Therefore, we assume that most adults in developed countries will have acquired this faculty before the onset of the pathology. However, it cannot be scientifically proven that all participants learned the drawing of geometric forms and the non-conscious acquiring of skills [46, 47] makes it difficult to gain insights into if and how the strategy of cube drawing has been acquired.

### Outlook

Our findings suggest that impaired functioning of retrograde procedural memory could be already detectable in a prodromal, non-motor stage of the disease and perhaps in the future be used as an early marker of Parkinson’s disease. Therefore, it would be of great interest to further investigate how this impairment evolves in relation to the disease progression in Parkinson’s disease. People with atypical parkinsonism have different and variable neuropsychological profiles. Future studies may compare the performance of retrograde procedural memory between the different forms of parkinsonism. Additionally, future research should validate the CUPRO evaluation system in independent Parkinson’s disease cohorts and with attention to possible relationships between impaired cube drawing performance in low and very low educated participants which we were not able to systematically test in our high-educated sample. Furthermore, future work should also provide a convergent test of the proposed evaluation tool with similar already existing assessments for the procedural memory, such as mirror tracing task and serial reaction time task.

## CONCLUSION

It is of great importance to get a deeper knowledge of the functioning of retrograde procedural memory, as the integrity of this part of the memory is crucial for a person’s ability to conduct routine activities of daily living, which ultimately serve to maintain independence. This study established a new tool to assess functioning of retrograde procedural memory and showed deficits in retrograde procedural memory in people with Parkinson’s disease compared with control subjects. The CUPRO evaluation system will not only fill the gap of techniques for reliably assessing functioning of retrograde procedural memory in clinical settings but may also help to identify valuable perspectives for future research.

## Supplementary Material

Supplementary Material
