# Supplementary material for: Retrograde Procedural Memory in Parkinson’s Disease: A Cross-Sectional, Case-Control Study
Source: J Parkinsons Dis. 2022 Apr 5;12(3):1013–22. doi: 10.3233/JPD-213081 (PMC9108589; doi:10.3233/JPD-213081)
Supplement: Supplementary Material [file jpd-12-jpd213081-s001.pdf]

Supplementary Material

Retrograde Procedural Memory in Parkinson's Disease: A Cross-Sectional, Case-Control Study

Supplementary Table 1. Correlations for the Intermediate Scores 1 and possible interfering factors

		N	Spearman – Correlation coefficient R	p
Visuo-constructive abilities	**Qualitative Scoring MMSE Pentagon Test**	54	- 0.103	0.462
Visuo-spatial abilities	**Benton Judgment of Line Orientation**	56	+ 0.058	0.249
Executive functions	**Frontal Assessment Battery**	73	+ 0.102	0.392
Visuo-constructive abilities	**Rey-Osterrieth Complex Figure - Copy**	34	+ 0.169	0.338
Planning functions	**Rey-Osterrieth Complex Figure - Type**	34	- 0.381	0.831

Supplementary Figure 1. Example of a cube drawing without a typical procedure. This participant would get a correct final result by copying the cube line by line, without using a pre-defined procedure.

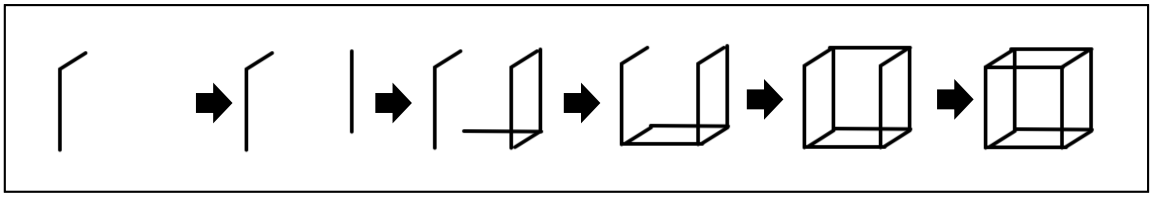

Supplementary Figure 2. Examples of cube drawings and their evaluation scores. (A) $IS_1 < 3$ and $IS_2 = 3$ or $IS_1 = 3$ and $IS_2 = 3$; (B) $IS_1 < 3$ and $IS_2 < 3$; (C) $IS_1 = 0$ and $IS_2 = 0$; (D) $IS_1 = 3$ and $IS_2 < 3$.

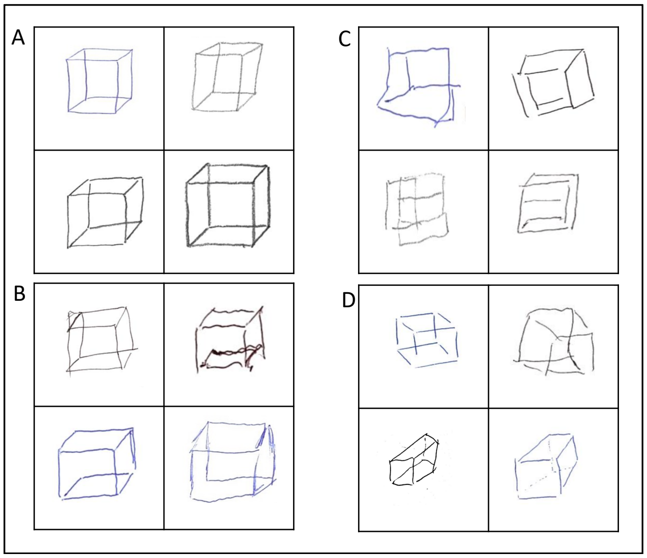

Further qualitative analyses revealed four possible scenarios for the final results of the cube copying:

(A) A correct final result of the cube drawing ($IS_2 = 3$), that was obtained by the application of a copying procedure ($IS_1 = 3$). Even when not using a typical copying procedure ($IS_1 < 3$) it was possible to get a correct final result ($IS_2 = 3$), by simply copying the cube line by line, continuously comparing their drawing with the figure without using a typical procedure (Supplementary Figure 1).

(B) A slightly incorrect final result of the cube drawing ($IS_2 < 3$), often characterized by the addition and/or omission of an element during the copying of the cube, in those who copied the cube line by line without a clear procedure ($IS_1 < 3$).

(C) An incorrect final result with almost no resemblance to the cube ($IS_2 = 0$) did not fulfill the above-described criteria and was therefore not correct ($IS_1 = 0$).

(D) A procedure was applied ($IS_1 = 3$) but the final result is wrong ($IS_2 < 3$). This observation is reflected by cubes that are wrongly oriented, wrong proportioned, or in mirror-representation.
